# Supplementary material for: Using null models to compare bacterial and microeukaryotic metacommunity assembly under shifting environmental conditions
Source: Sci Rep. 2020 Feb 12;10:2455. doi: 10.1038/s41598-020-59182-1 (PMC7016149; doi:10.1038/s41598-020-59182-1)
Supplement: Supplementary file 1 — Supplementary Information. [file 41598_2020_59182_MOESM1_ESM.pdf]

Supplementary Data and Methods for: Using null models to compare bacterial and microeukaryotic metacommunity assembly under shifting environmental conditions

Máté Vass*, Anna J. Székely, Eva S. Lindström, Silke Langenheder

Department of Ecology and Genetics/Limnology, Uppsala University, Sweden
*corresponding author's e-mail address: mate.vass@ebc.uu.se

Sampling site

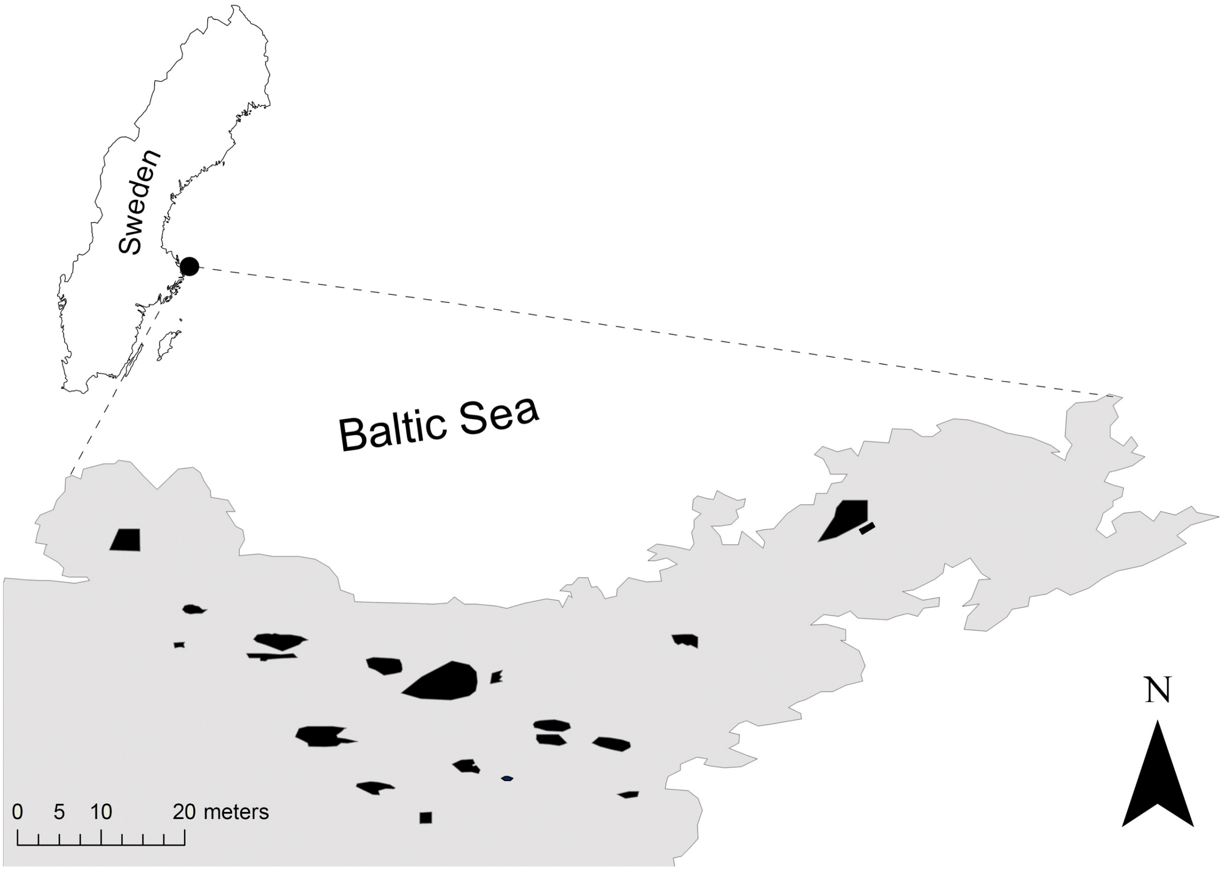

Figure S1. Sampled rock pools along the Baltic Sea coast on the island of Gräsö, Sweden. Copyright by Lantmäteriet Gävle (2012): Permission i2012/921.

Meteorological conditions

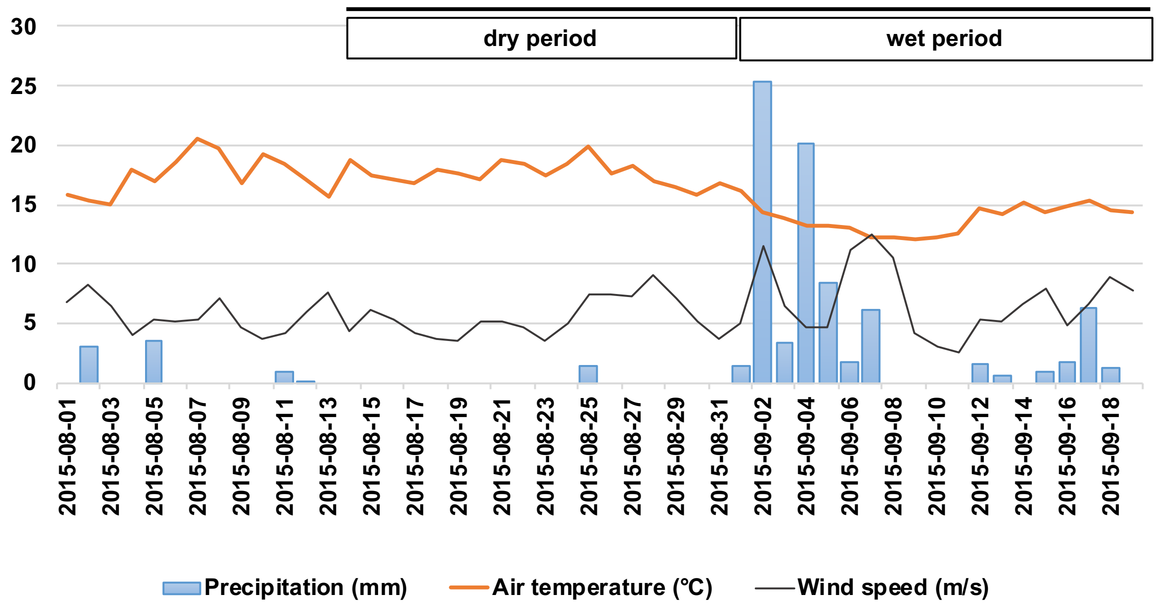

Figure S2. Daily total precipitation (mm), daily mean air temperature (°C) and daily mean wind speed (m/s) conditions at the Örskär meteorological station obtained from the Swedish Meteorological and Hydrological Institute (SMHI). The black bar refers to the study period.

Taxonomic classification and distribution of OTUs found in the rock pools

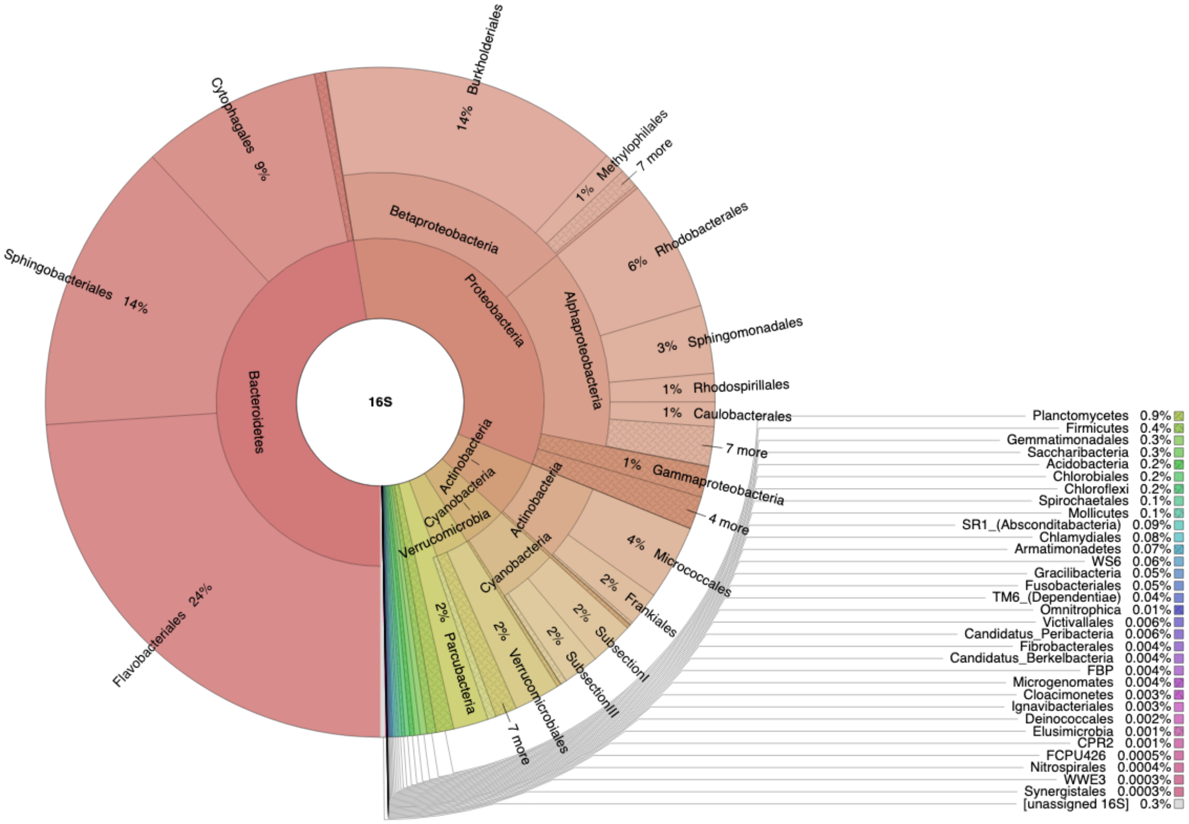

Figure S3. Taxonomic classification and distribution of bacterial OTUs (total of 4,587 OTUs)

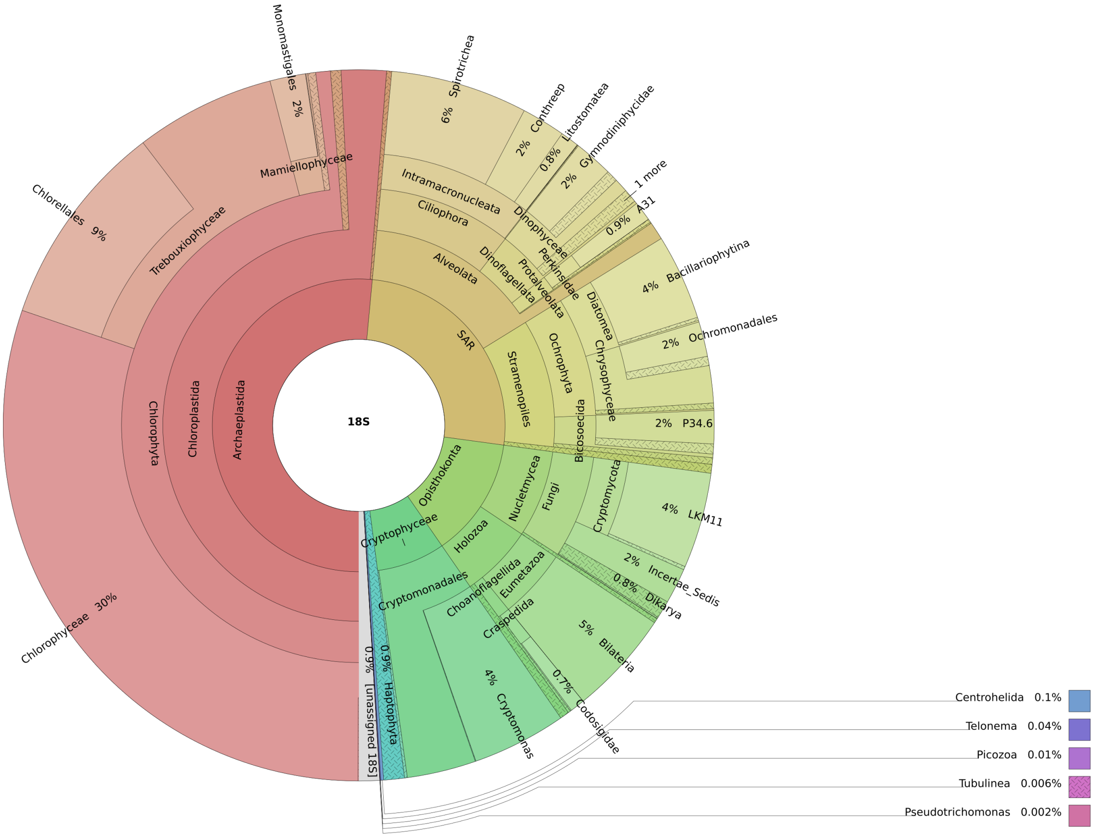

Figure S4. Taxonomic classification and distribution of microeukaryotic OTUs (total of 1,336 OTUs)

Phylogenetic signals

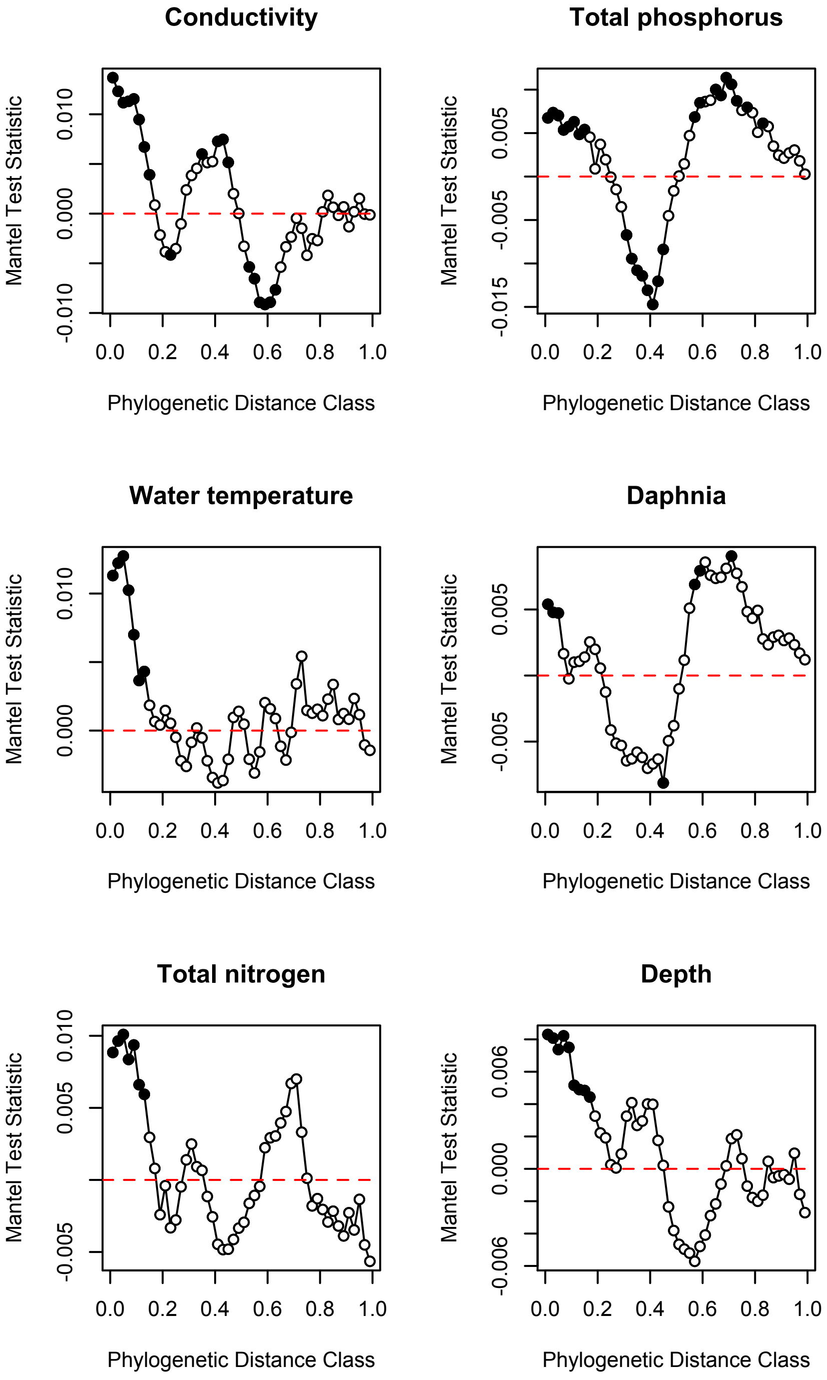

Figure S5. Phylogenetic Mantel correlograms showing significant phylogenetic signals across short phylogenetic distances (PD) in bacterioplankton communities. Closed dots denote significant correlations, relating between-OTU niche differences to between-OTU PDs across a given PD. Estimates of optimal OTU environmental niches were calculated for conductivity, total phosphorus, water temperature, *Daphnia* abundance, total nitrogen and rock pool depth.

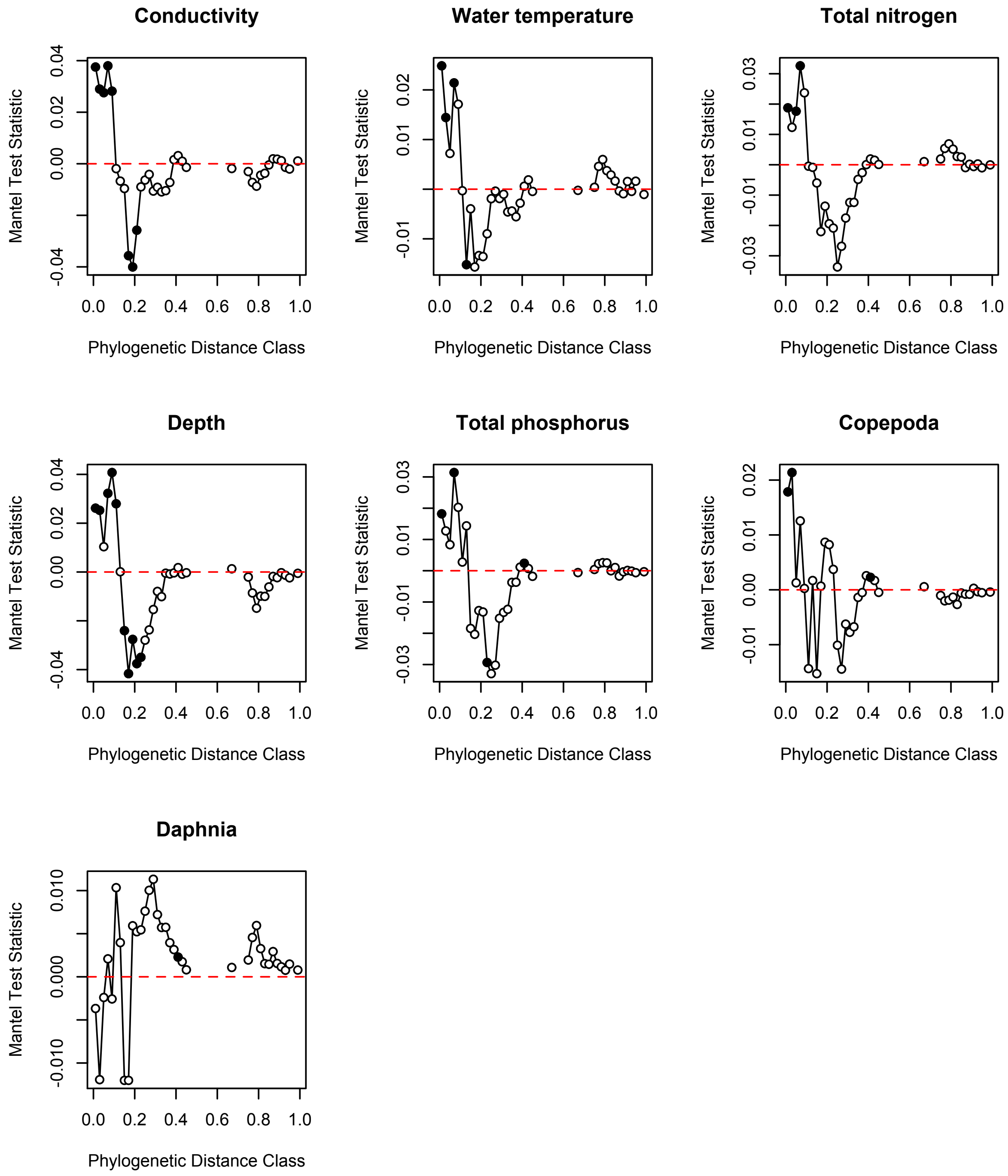

Figure S6. Phylogenetic Mantel correlograms showing significant phylogenetic signals across short phylogenetic distances (PD) in microeukaryotic communities (except in case of *Daphnia* abundance). Closed dots denote significant correlations, relating between-OTU niche differences to between-OTU PDs across a given PD. Estimates of optimal OTU environmental niches were calculated for conductivity, water temperature, total nitrogen, rock pool depth, total phosphorus, *Copepoda* and *Daphnia* abundances.

Evidences for shifting environmental conditions in the rock pools during the study period and its effect on bacterial and microeukaryotic community compositions

Table S1. Characteristic differences of rock pools between the dry and wet period. A Kruskal-Wallis test was used to compare the means, while Levene's test was used to compare the homogeneity of variance for each environmental variable. Significant values ($p < 0.05$) are in bold.

	**Mean**		**Variance**	
	chi-squared	*P*	*F*	*P*
Conductivity	4.972	**0.0257**	3.586	0.059
Copepoda abundance	31.525	**<0.0001**	44.68	**<0.0001**
Daphnia abundance	5.536	**0.0186**	24.43	**<0.0001**
Depth	49.047	**<0.0001**	0.335	0.563
Total nitrogen	109.4	**<0.0001**	16.319	**<0.0001**
Total phosphorus	70.002	**<0.0001**	24.96	**<0.0001**
Water temperature	143.09	**<0.0001**	32.42	**<0.0001**

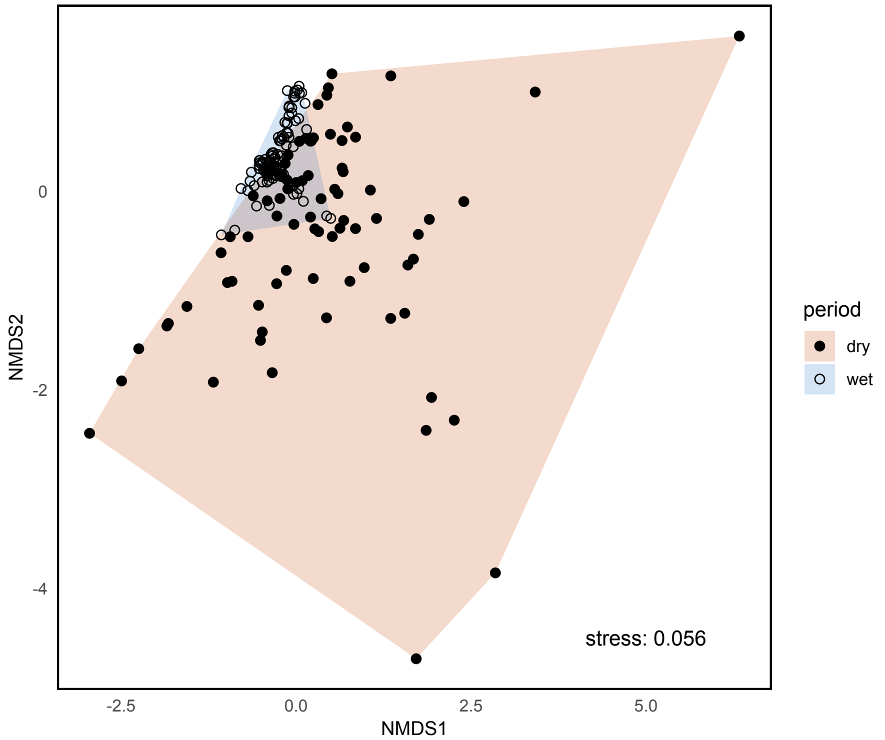

Figure S7. NMDS plot showing the changes in environmental conditions (PERMANOVA: F = 31.07, $R^2 = 0.15$, $p = 0.001$; PERMDISP: F = 79.58, $p < 0.001$) in relation to the dry and wet period based on Euclidean distances.

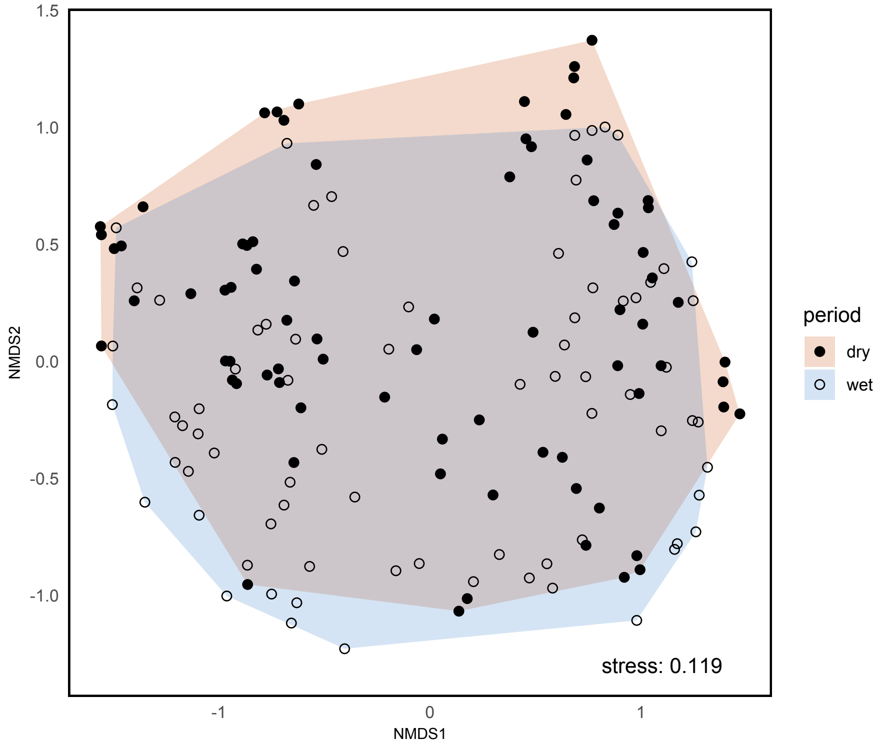

Figure S8. NMDS plot showing the changes in bacterial communities (PERMANOVA: F = 3.68, $R^2 = 0.024$, $p = 0.001$; PERMDISP: F = 0.558, $p = 0.456$) in relation to the dry and wet period based on Bray-Curtis dissimilarities.

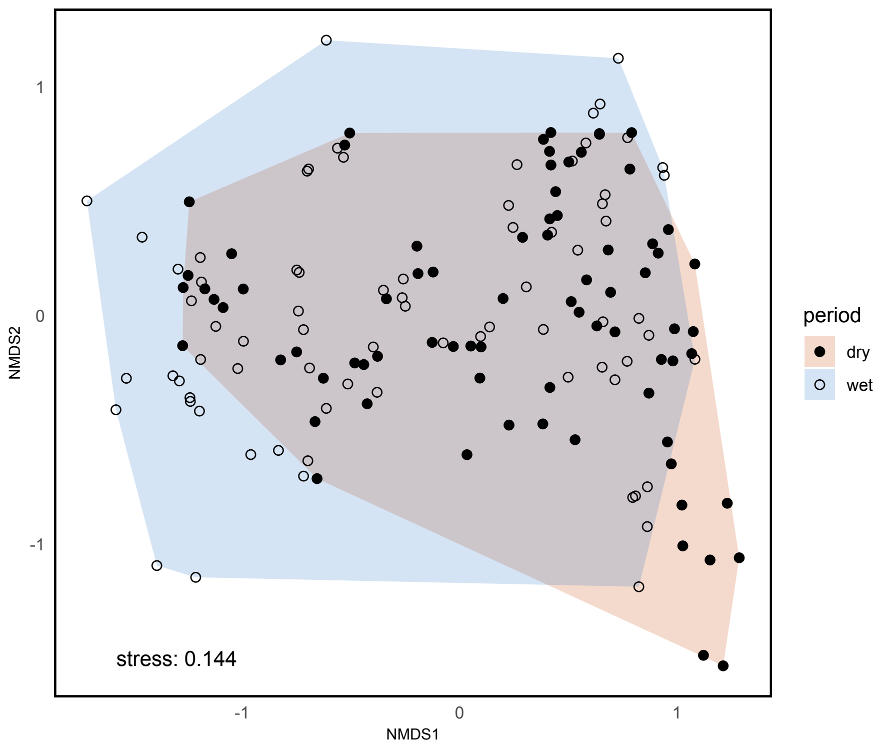

Figure S9. NMDS plot showing the changes in microeukaryotic communities (PERMANOVA: F = 5.26, R^2 = 0.034, p = 0.001; PERMDISP: F = 1.639, p = 0.202) in relation to the dry and wet period based on Bray-Curtis dissimilarities.

Table S2. Difference in variation of environmental conditions, bacterial and microeukaryotic compositions between the dry and wet period in each rock pool based on permutational multivariate analysis of variance (PERMANOVA) using Euclidean distances (for the environmental data) and Bray-Curtis distances (for the Hellinger-transformed OTUs datasets) with 999 permutations. Significant values (p < 0.05) are in bold.

Rock pool ID		*Environment*						*Bacterial composition*						*Microeukaryotic composition*					
		**df**	**SS**	**MeanSqs**	**F-value**	**R^2**	**Pr(>F)**	**df**	**SS**	**MeanSqs**	**F-value**	**R^2**	**Pr(>F)**	**df**	**SS**	**MeanSqs**	**F-value**	**R^2**	**Pr(>F)**
**RP1**	dry/wet	1	10.755	10.755	7.231	0.475	**0.005**	1	0.251	0.251	1.204	0.131	0.275	1	0.861	0.861	4.675	0.400	**0.011**
	Residuals	8	11.900	1.487		0.525		8	1.666	0.208		0.869		7	1.289	0.184		0.600	
	Total	9	22.655			1.000		9	1.916			1.000		8	2.150			1.000	
**RP2**	dry/wet	1	10.031	10.031	7.680	0.490	**0.011**	1	0.37	0.37	1.301	0.140	0.195	1	0.822	0.822	5.140	0.423	**0.02**
	Residuals	8	10.449	1.306		0.510		8	2.272	0.284		0.860		7	1.120	0.160		0.577	
	Total	9	20.480			1.000		9	2.642			1.000		8	1.942			1.000	
**RP3**	dry/wet	1	6.961	6.961	4.477	0.359	**0.024**	1	0.406	0.406	1.541	0.162	0.168	1	0.521	0.521	2.404	0.231	**0.041**
	Residuals	8	12.440	1.555		0.641		8	2.105	0.263		0.838		8	1.733	0.217		0.769	
	Total	9	19.401			1.000		9	2.511			1.000		9	2.254			1.000	
**RP4**	dry/wet	1	7.704	7.704	4.613	0.366	**0.023**	1	0.585	0.585	2.219	0.241	**0.043**	1	0.785	0.785	3.436	0.300	**0.014**
	Residuals	8	13.362	1.670		0.634		7	1.847	0.264		0.759		8	1.828	0.228		0.700	
	Total	9	21.067			1.000		8	2.432			1.000		9	2.613			1.000	
**RP5**	dry/wet	1	9.395	9.395	5.305	0.399	**0.007**	1	0.833	0.833	4.095	0.339	**0.012**	1	0.585	0.585	2.578	0.269	**0.012**
	Residuals	8	14.169	1.771		0.601		8	1.628	0.203		0.661		7	1.589	0.227		0.731	
	Total	9	23.565			1.000		9	2.461			1.000		8	2.174			1.000	
**RP7**	dry/wet	1	4.229	4.229	5.308	0.399	**0.008**	1	0.35	0.35	1.145	0.141	0.29	1	0.949	0.949	6.321	0.441	**0.008**
	Residuals	8	6.374	0.797		0.601		7	2.139	0.306		0.859		8	1.201	0.150		0.559	
	Total	9	10.604			1.000		8	2.488			1.000		9	2.150			1.000	
**RP8**	dry/wet	1	12.431	12.431	11.902	0.630	**0.007**	1	0.467	0.467	1.641	0.190	0.053	1	0.637	0.637	2.721	0.254	**0.008**
	Residuals	7	7.312	1.045		0.370		7	1.992	0.285		0.810		8	1.872	0.234		0.746	
	Total	8	19.743			1.000		8	2.459			1.000		9	2.509			1.000	
**RP11**	dry/wet	1	3.706	3.706	4.929	0.413	**0.012**	1	0.303	0.303	1.285	0.138	0.174	1	0.598	0.598	4.165	0.373	0.055
	Residuals	7	5.263	0.752		0.587		8	1.889	0.236		0.862		7	1.005	0.144		0.627	
	Total	8	8.970			1.000		9	2.192			1.000		8	1.603			1.000	
**RP12**	dry/wet	1	6.063	6.063	5.823	0.454	**0.011**	1	0.26	0.26	0.848	0.108	0.515	1	0.863	0.863	6.750	0.458	**0.017**
	Residuals	7	7.289	1.041		0.546		7	2.146	0.307		0.892		8	1.023	0.128		0.542	
	Total	8	13.351			1.000		8	2.407			1.000		9	1.886			1.000	
**RP13**	dry/wet	1	8.837	8.837	9.480	0.575	**0.014**	1	0.412	0.412	2.16	0.236	**0.018**	1	0.895	0.895	5.415	0.404	**0.015**
	Residuals	7	6.525	0.932		0.425		7	1.335	0.191		0.764		8	1.323	0.165		0.596	
	Total	8	15.362			1.000		8	1.747			1.000		9	2.218			1.000	
**RP15**	dry/wet	1	7.988	7.988	5.703	0.449	**0.006**	1	0.415	0.415	2.213	0.240	**0.037**	1	1.076	1.076	8.204	0.540	**0.004**
	Residuals	7	9.804	1.401		0.551		7	1.312	0.187		0.760		7	0.918	0.131		0.460	
	Total	8	17.792			1.000		8	1.727			1.000		8	1.994			1.000	
**RP16**	dry/wet	1	7.902	7.902	9.554	0.577	**0.01**	1	0.454	0.454	1.486	0.157	0.115	1	0.602	0.602	3.248	0.289	**0.008**
	Residuals	7	5.790	0.827		0.423		8	2.443	0.305		0.843		8	1.482	0.185		0.711	
	Total	8	13.692			1.000		9	2.897			1.000		9	2.084			1.000	
**RP17**	dry/wet	1	4.282	4.282	2.196	0.239	**0.014**	1	0.47	0.47	1.638	0.170	0.091	1	0.672	0.672	4.843	0.447	**0.017**
	Residuals	7	13.649	1.950		0.761		8	2.293	0.287		0.830		6	0.832	0.139		0.553	
	Total	8	17.931			1.000		9	2.763			1.000		7	1.504			1.000	
**RP18**	dry/wet	1	6.223	6.223	4.835	0.409	**0.005**	1	0.43	0.43	1.945	0.196	**0.027**	1	0.927	0.927	5.704	0.449	**0.003**
	Residuals	7	9.008	1.287		0.591		8	1.769	0.221		0.804		7	1.138	0.163		0.551	
	Total	8	15.231			1.000		9	2.2			1.000		8	2.065			1.000	
**RP19**	dry/wet	1	4.719	4.719	4.565	0.395	**0.009**	1	0.56	0.56	3.139	0.310	**0.006**	1	1.066	1.066	8.329	0.510	**0.005**
	Residuals	7	7.236	1.034		0.605		7	1.25	0.179		0.690		8	1.024	0.128		0.490	
	Total	8	11.955			1.000		8	1.81			1.000		9	2.090			1.000	
**RP20**	dry/wet	1	5.855	5.855	3.961	0.361	**0.013**	1	0.608	0.608	3.049	0.303	**0.018**	1	0.676	0.676	2.508	0.239	**0.009**
	Residuals	7	10.346	1.478		0.639		7	1.396	0.199		0.697		8	2.157	0.270		0.761	
	Total	8	16.201			1.000		8	2.004			1.000		9	2.833			1.000	

Table S3. Difference in variation of environmental conditions, bacterial and microeukaryotic compositions between the dry and wet period in each rock pool based on multivariate homogeneity of group dispersions (PERMDISP) using Euclidean distances (for the environmental data) and Bray-Curtis distances (for the Hellinger-transformed OTUs datasets). Significant values ($p < 0.05$) are in bold.

Rock pool ID		*Environment*					*Bacterial composition*					*Microeukaryotic composition*				
		**df**	**SS**	**MeanSqs**	**F-value**	**Pr(>F)**	**df**	**SS**	**MeanSqs**	**F-value**	**Pr(>F)**	**df**	**SS**	**MeanSqs**	**F-value**	**Pr(>F)**
**RP1**	dry/wet	1	0.069	0.069	0.077	0.789	1	0.004	0.004	0.167	0.694	1	0.046	0.046	1.788	0.223
	Residuals	8	7.194	0.899			8	0.190	0.024			7	0.178	0.025		
**RP2**	dry/wet	1	4.434	4.434	94.698	**<0.001**	1	0.002	0.002	0.145	0.713	1	0.078	0.078	3.528	0.102
	Residuals	8	0.375	0.047			8	0.116	0.014			7	0.155	0.022		
**RP3**	dry/wet	1	1.161	1.161	3.643	0.093	1	0.011	0.011	0.389	0.550	1	0.004	0.004	0.192	0.673
	Residuals	8	2.551	0.319			8	0.230	0.029			8	0.181	0.023		
**RP4**	dry/wet	1	2.331	2.331	3.577	0.095	1	0.010	0.010	0.665	0.442	1	0.001	0.001	0.023	0.882
	Residuals	8	5.214	0.652			7	0.107	0.015			8	0.229	0.029		
**RP5**	dry/wet	1	0.127	0.127	0.128	0.730	1	0.026	0.026	4.409	0.069	1	0.001	0.001	0.181	0.683
	Residuals	8	7.986	0.998			8	0.046	0.006			7	0.044	0.006		
**RP7**	dry/wet	1	1.021	1.021	3.450	0.100	1	0.001	0.001	0.017	0.900	1	0.007	0.007	0.260	0.624
	Residuals	8	2.368	0.296			7	0.234	0.033			8	0.229	0.029		
**RP8**	dry/wet	1	1.555	1.555	7.378	**0.030**	1	0.000	0.000	0.011	0.920	1	0.009	0.009	0.703	0.426
	Residuals	7	1.475	0.211			7	0.090	0.013			8	0.106	0.013		
**RP11**	dry/wet	1	0.878	0.878	3.260	0.114	1	0.017	0.017	0.334	0.579	1	0.076	0.076	2.633	0.149
	Residuals	7	1.885	0.269			8	0.418	0.052			7	0.201	0.029		
**RP12**	dry/wet	1	0.061	0.061	0.113	0.747	1	0.001	0.001	0.014	0.908	1	0.051	0.051	4.990	0.056
	Residuals	7	3.785	0.541			7	0.426	0.061			8	0.082	0.010		
**RP13**	dry/wet	1	1.538	1.538	17.674	**0.004**	1	0.001	0.001	0.027	0.875	1	0.003	0.003	0.095	0.765
	Residuals	7	0.609	0.087			7	0.144	0.021			8	0.254	0.032		
**RP15**	dry/wet	1	1.653	1.653	3.059	0.124	1	0.013	0.013	1.405	0.275	1	0.006	0.006	0.840	0.390
	Residuals	7	3.781	0.540			7	0.063	0.009			7	0.054	0.008		
**RP16**	dry/wet	1	1.459	1.459	7.391	**0.030**	1	0.001	0.001	0.040	0.846	1	0.038	0.038	2.945	0.125
	Residuals	7	1.382	0.197			8	0.186	0.023			8	0.104	0.013		
**RP17**	dry/wet	1	0.167	0.167	0.139	0.720	1	0.008	0.008	1.247	0.297	1	0.005	0.005	0.407	0.547
	Residuals	7	8.388	1.198			8	0.050	0.006			6	0.067	0.011		
**RP18**	dry/wet	1	3.054	3.054	47.051	**<0.001**	1	0.035	0.035	1.793	0.217	1	0.067	0.067	2.620	0.150
	Residuals	7	0.454	0.065			8	0.155	0.019			7	0.178	0.025		
**RP19**	dry/wet	1	1.777	1.777	4.377	0.075	1	0.031	0.031	2.099	0.191	1	0.041	0.041	2.837	0.131
	Residuals	7	2.842	0.406			7	0.102	0.015			8	0.115	0.014		
**RP20**	dry/wet	1	2.775	2.775	3.593	0.100	1	0.020	0.020	0.508	0.499	1	0.014	0.014	1.507	0.255
	Residuals	7	5.408	0.773			7	0.275	0.039			8	0.075	0.009		

Results of the elements of metacommunity structure (EMS) analyses

Table S4. Results of EMS analysis for bacterial and microeukaryotic communities based on fixed-proportional null models performed separately on matrices ranked based on the first ordination (primary) axis extracted via reciprocal averaging. *Abs*: number of embedded absences, *Turn*: number of replacements. Interpretations follow Presley et al. [1]. Significant results (p < 0.05) are in bold.

Bacterioplankton

	Sampling	Percent	Coherence					Turnover					Clumping			Metacommunity
	dates	inertia	Abs	z	p	sim.Mean	sim.Var	Turn	z	p	sim.Mean	sim.Var	index	p	df	type
Primary axis	2015-08-14	11.6%	21705	-0.220	0.825	21529.6	795.5	7147070	-6.564	**<0.0001**	6566729.5	88408.9	1.156	**<0.0001**	12	Random
	2015-08-18	12.1%	23389	-1.555	0.120	22061.2	853.8	8101347	-1.588	0.112	7936575.0	103784.0	1.097	**<0.0001**	11	Random
	2015-08-22	10.9%	33565	-2.359	**0.018**	30909.2	1125.7	13866336	-4.329	**<0.0001**	13141599.3	167417.5	1.303	**<0.0001**	13	Checkerboards
	2015-08-26	11.1%	27107	1.787	0.074	28800.8	947.9	12817243	-10.033	**<0.0001**	11492551.9	132036.7	1.156	**<0.0001**	13	Random
	2015-08-30	10.5%	28358	1.244	0.213	29656.5	1043.8	13779751	-12.706	**<0.0001**	11959169.3	143282.6	1.281	**<0.0001**	13	Random
	2015-09-03	12.1%	33743	-0.075	0.940	33657.4	1136.3	20594763	-9.206	**<0.0001**	18575990.0	219277.5	1.109	**<0.0001**	13	Random
	2015-09-07	11.6%	27946	-0.493	0.622	27477.0	951.4	12822665	-9.318	**<0.0001**	11527035.1	139050.0	1.135	**<0.0001**	12	Random
	2015-09-11	12.7%	21750	3.976	**<0.0001**	25194.9	866.3	11178647	-17.021	**<0.0001**	9238071.1	114009.0	1.067	**<0.0001**	12	Nested - Clumped species loss
	2015-09-15	13.4%	22805	1.483	0.138	24097.7	871.7	9307139	-12.374	**<0.0001**	8131338.5	95020.3	1.163	**<0.0001**	12	Random
	2015-09-19	12.3%	17095	0.718	0.473	17635.0	752.4	4750609	-5.066	**<0.0001**	4416531.7	65951.5	1.157	**<0.0001**	11	Random

Microeukaryotes

	Sampling	Percent	Coherence					Turnover					Clumping			Metacommunity
	dates	inertia	Abs	z	p	sim.Mean	sim.Var	Turn	z	p	sim.Mean	sim.Var	index	p	df	type
Primary axis	2015-08-14	12.6%	4593	-1.823	0.068	4213.8	208.0	346150	0.163	0.870	347921.7	10854.6	1.187	**<0.0001**	10	Random
	2015-08-18	11.1%	7951	**-2.484**	**0.013**	7264.8	276.2	921033	2.147	**0.032**	989418.8	31858.2	1.128	**<0.0001**	13	Checkerboards
	2015-08-22	13.6%	8291	-1.372	0.170	7850.7	321.0	1159339	2.200	**0.028**	1232054.0	33050.4	1.138	**<0.0001**	13	Random
	2015-08-26	12.0%	6803	-1.280	0.200	6455.0	271.8	766156	2.940	**0.003**	836760.9	24013.7	1.124	**<0.0001**	12	Random
	2015-08-30	11.8%	8068	-1.485	0.138	7642.9	286.2	1171495	-1.833	0.067	1117742.8	29318.8	1.064	**<0.0001**	13	Random
	2015-09-03	12.7%	9842	**-2.440**	**0.015**	8951.0	365.2	1618172	3.406	**0.001**	1769959.3	44569.6	1.140	**<0.0001**	12	Checkerboards
	2015-09-07	13.4%	7779	1.125	0.260	8159.6	338.2	1437851	-8.138	**0.000**	1193279.8	30051.5	1.195	**<0.0001**	12	Random
	2015-09-11	10.6%	8767	-0.899	0.369	8473.5	326.4	1440551	-7.356	**0.000**	1213772.3	30828.4	1.129	**<0.0001**	13	Random
	2015-09-15	11.5%	7250	-0.237	0.813	7183.4	281.1	890580	-3.938	**0.000**	811100.0	20181.0	1.100	**<0.0001**	12	Random
	2015-09-19	11.5%	7001	-0.486	0.627	6857.7	294.6	860170	0.016	0.987	860478.6	19263.6	1.092	**<0.0001**	12	Random

Appendix 1

Sequence processing

Raw sequence data were analyzed and sequences clustered into 97 % similarity OTUs according to the UPARSE pipeline [2], as described on the BILS website for using UPARSE on the UPPMAX cluster (available with all scripts at: https://wiki.bils.se/wiki/Running_the_Uparse_pipeline_at_the_UPPMAX_cluster).
We further assigned taxonomy to the representative 16S and 18S OTUs using the SSU Ref NR 99 v119 SILVA database [3]. Chloroplast OTUs and OTUs that were unassigned taxonomically or represented by less than 10 sequences were discarded. To generate an equal number of sequences per sample the OTU table was subsampled based on the sample with the lowest number of reads (4 940 reads/sample in the bacterial dataset, 3 429 reads/sample in the microeukaryotic dataset). A total of 7 767 202 quality controlled 16S rRNA sequencing reads were acquired. After OTU clustering (sample concatenation, dereplication and sorting) 5 291 921 sequences were taxonomically assigned (with a 95 % sequence identity threshold). After removal of non-bacterial sequences and subsampling, a total of 4 587 OTUs were retained for the bacterial communities. In the 18S rRNA dataset, 6 349 385 good-quality reads were gained and 3 776 690 sequences were taxonomically assigned (with 95% sequence identity threshold) after OTU clustering. After removal of non-eukaryotic OTUs and subsampling, the microeukaryotic dataset consisted of 1 336 OTUs. The taxonomic distribution of reads for both datasets was visualized with Krona (http://sourceforge.net/projects/krona).

Phylogenetic tree building

To construct phylogenetic trees, first, the representative sequences of each OTU were aligned to the Greengenes core reference alignment [4] in the case of bacteria, and to the SILVA 128 core reference alignment [3] in the case of microeukaryotes using PyNAST [5] in MacQIIME v1.9.1 [6]. To remove gaps and highly variable regions, the alignments were filtered using a Lane mask [7] and were then used to construct approximate maximum-likelihood phylogenetic trees with FastTree v2.1.3.

Appendix 2

Detailed description of the method of elements of metacommunity structure (EMS)

We examined coherence where statistical significance of the number of embedded absences (i.e., the interruptions in the species distributions) was determined with z-tests resulting in z-value of coherence. Non-significant z-values (no coherence) suggest randomly structured assemblages. z-values that are significantly lower than in the null distribution (negative coherence) suggest checkerboard patterns due to competitive exclusions. Finally, z-values that are significantly higher compared to the null model distribution (positive coherence) indicate that assemblages are structured along environmental gradients, either individualistically or by coherent units of species that respond similarly to the changes in the environment.

In the next step we, therefore, examined species turnover based on z-tests to depict the character of these gradual changes. If turnover was significantly higher than expected by chance, this is indicative of species sorting along environmental gradients and boundary clumping based on Morisita's index was used to describe whether individualistic (Gleasonian pattern, replacement of individual species along the gradient), synchronous (Clementsian pattern, replacement of groups of species along the gradient) or evenly spaced species turnover was important. If turnover is significantly lower than expected by chance, the metacommunity had nested subsets in which species in less diverse communities are subsets of those in more diverse communities [8].

In addition to the original framework by Leibold & Mikkelson [8], Presley et al. [1] refined the evaluation of the analyses of metacommunity structure. Following their suggestions, we could distinguish three special cases when nested subsets occur as a result of significantly lower turnover. For example, random, clumped (clumped boundaries), or a hyperdispersed (no clumping) species loss can be distinguished by the assessments of range boundary clumping (Morisita's index). Each of these three nested patterns is analogous to Gleasonian, Clementsian and evenly spaced structure.

References

1. Presley, S. J., Higgins, C. L. & Willig, M. R. A comprehensive framework for the evaluation of metacommunity structure. *Oikos* **119**, 908–917 (2010).
2. Edgar, R. C. UPARSE: highly accurate OTU sequences from microbial amplicon reads. *Nat. Methods* **10**, 996–998 (2013).
3. Quast, C. *et al.* The SILVA ribosomal RNA gene database project: Improved data processing and web-based tools. *Nucleic Acids Res.* **41**, (2013).
4. DeSantis, T. Z. *et al.* Greengenes, a chimera-checked 16S rRNA gene database and workbench compatible with ARB. *Appl. Environ. Microbiol.* **72**, 5069–5072 (2006).
5. Caporaso, J. G. *et al.* PyNAST: A flexible tool for aligning sequences to a template alignment. *Bioinformatics* **26**, 266–267 (2010).
6. Caporaso, J. G. *et al.* QIIME allows analysis of high-throughput community sequencing data. *Nature Methods* **7**, 335–336 (2010).
7. Price, M. N., Dehal, P. S. & Arkin, A. P. FastTree 2 - Approximately maximum-likelihood trees for large alignments. *PLoS One* **5**, (2010).
8. Leibold, M. A. & Mikkelson, G. M. Coherence, species turnover, and boundary clumping: elements of meta-community structure. *Oikos* **97**, 237–250 (2002).
